# Laryngology and Rhinology

**Published:** 1899-12

**Authors:** Barclay J. Baron


### LARYNGOLOGY AND RHINOLOGY.

Dr. Dundas Grant[2] drew attention to a very important matter, at the Portsmouth meeting of the British Medical Association, when he pointed out that amongst many **causes of headache** nasal and aural disease must be remembered. As regards the forms of nasal disease that may give rise to headache, adenoid vegetations in the naso-pharynx and hypertrophy of the middle turbinated body are the most frequent. Then come disease of the accessory sinuses, antrum of Highmore, frontal, sphenoidal, and ethmoidal. Operative measures for the cure of the nasal mischief cured the headache.

Hajek[3] alludes to the same matter, and says that the headache

[1] *Loc. cit.* [2] *J. Laryngol.*, 1899, xiv. 452.

[3] *München. med. Wchnschr,* 1899, xlvi. 336.

may depend directly on the nasal disease and disappears with its cure, or the nasal condition predisposes to headache. Apart from ulcerative conditions of the nose, disease of the accessory sinuses and hypertrophic changes of the nasal mucous membrane come under consideration. In disease of the sinuses it may be of a neuralgic nature or of an indefinite character (frontal, vertical, feeling of pressure or of numbness) or hemicrania. The pain in acute empyema of the antrum or frontal sinus may be in the infra-orbital, superior dental or supra-orbital nerve. It varies in intensity at different times. It is more apt to be neuralgic in disease of the frontal sinus than in that of the antrum, and in the acute stage of frontal sinus mischief it is intense. It may be absent in chronic empyema of the antrum, and also, but less frequently, in that of the frontal sinus. It is aggravated by coryza, mental or physical disturbance, and abuse of alcohol. Simple hypertrophy of the turbinated bodies is rarely a cause of headache; but where the septum is also deflected or thickened so that pressure can take place, then patients complain of heaviness in the head and pressure at the root of the nose.

A discussion on the diagnosis and treatment of **chronic empyema of the frontal sinus** took place at the same meeting.[1] The discussion was opened by Mr. Charters Symonds, of London, and Dr. E. J. Moure, of Bordeaux.

Mr. Charters Symonds divided the cases into three groups:—(i.) Those in which there is purulent discharge from the nose, with, as a rule, formation of polypi. (ii.) Those in which there is distension of the sinus without nasal discharge. (iii.) Those in which there is distension of sinus, together with nasal discharge of pus. Attention was chiefly given to the diagnosis of the first class of cases, as the class most frequently coming before the rhinologist. He laid stress upon the fact that, whenever pus was seen amongst or around polypi, suppuration of one or more of the sinuses was indicated. He considered the pus to be the cause of the polypi, and to explain the frequent recurrence of polypi when the pus itself had not been traced to its origin. Where the polypi were numerous, it was impossible to say from which sinus the pus was coming, but he held that where they were very numerous, and there was much pus, with a foul odour, the maxillary antrum was certainly involved, with or without the frontal sinus. In the pure frontal cases the polypi were less numerous, the granulations fewer, and the pus as a rule inodorous; in these cases also there was no pain.

Dr. Moure said that although habitually associated with empyema of the antrum, it occurs alone. Probable signs were: unilateral discharge of pus, seen on rhinoscopic examination

[1] *J. Laryngol.*, 1899, xiv. 469.

after the antrum has been thoroughly cleaned by irrigation; growths in the upper part of the infundibulum, in the direction of the naso-frontal canal, with dilatation of this canal, giving free access to the sinus; supra-orbital pains, spontaneous or on pressure. As certain signs he mentioned temporary or permanent swelling over the frontal sinus, or the presence of a fistula in that region; the flowing away of pus after irrigation, when that is possible in the frontal sinus; darkness on transillumination as compared with the opposite side. Absence of the sinus, fortunately rare, also gives rise to this sign.

The differential diagnosis between frontal empyema and that of the anterior ethmoidal cells may be difficult, but injection and transillumination will generally solve the difficulty. Ethmoidal growths are usually situated further back than those coming from the frontal sinus.

As regards treatment, there was a balance of opinion in favour of intranasal methods, and removal of the anterior portion of the middle turbinated body was held to be often of much value. Breaking into the sinus from the nose was considered not to be devoid of risk, and for bad cases an external operation and securing free drainage into the nose is necessary.

Dr. Milligan,[1] writing on the same subject, lays much stress upon the presence of pain when pressure is made just under the supra-orbital arch over the floor of the sinus and in a direction upwards and inwards, as a diagnostic sign. Transillumination is of value; and when pus is seen to proceed from the region of the infundibulum and there is opacity over the area of the frontal sinus, the presumption is certainly in favour of the presence of a frontal empyema. An accurate diagnosis is very difficult to arrive at, and opening and inspecting the sinus is often the only way of verifying it.

Mr. A. J. Brady[2] relates a case of **muco-purulent catarrh of the antrum of Highmore** simulating post-nasal catarrh, in which there was no discharge from the anterior nares after inversion of the head, nor was any to be seen in this situation. Posterior rhinoscopy revealed a thin rope of muco-pus issuing below the posterior end of the right middle turbinal, and extending above the right Eustachian cushion on to the post-pharyngeal wall. The antrum was opened and found to contain pus and polypoid tissue. The reason assigned for the direction of the flow of muco-pus backwards into the naso-pharnyx is either some local abnormality, or more probably the thickness of the discharge which clings to the membrane uninfluenced by gravitation. Ciliary motion may have something to do with its backward course.

[1] *J. Laryngol.*, 1899, xiv. 568. [2] *Ibid.*, 565.

Villy, writing on vomiting and cardiac failure in connection with **diphtheria**[1] states his objections to the "paralysis theory." In the present state of our knowledge it is almost impossible to prove or disprove the existence of cardiac paralysis of nervous origin, but he brings forward a good deal of clinical and pathological evidence to establish the following points as a summary of the pathological changes found. Out of 15 cases, the stomach showed fatty degeneration of the glandular epithelium in all, an excess of leucocytes in the mucous and submucous layers in all, hemorrhages in the mucous membrane in 8. The heart showed fatty degeneration in 14 cases, granular changes in the fibres and indistinctness or loss of their striation in 15 cases, hemorrhages in 7 cases. *Ante-mortem* clot was found in its cavities 8 times. The day of disease on which death occurred ranged from the 3rd to 40th. The degenerative changes of the heart and stomach, therefore, begin early in the disease and persist for a prolonged period.

From the clinical evidence it may be deduced that: (1) Signs of heart-failure are much more common than are paralyses in other parts, and have an earlier date of onset; (2) They are such as may be ascribed to muscular failure; (3) When vomiting occurs, heart-failure generally follows; (4) The date of vomiting and heart-failure is distinctly anterior to that of paralysis in other parts.

From the pathological data it appears that: (1) Evidence of degeneration and inflammation of the mucous membrane of the stomach is constantly present, and is often accompanied by hemorrhages; (2) The heart-muscle is constantly found to be in a degenerated condition; hemorrhages are commonly present.

Goodale[2] examined 16 cases of **acute tonsillitis** which had been inflamed for three or four days, and which were of non-diphtheritic origin. Sections of the excised glands show a diffuse inflammation of the parenchyma of the organ, appearing in the form of an increased proliferation of lymphoid cells, and of the endothelioid cells of the reticulum, due probably to the absorption of toxin formed in the crypts. While bacteria are rarely demonstrable in the tonsillar tissue in cases characterised by purely proliferative lesions, yet at times infection of the follicles occurs, giving rise to circumscribed suppuration and the formation of abscesses, which eventually discharge into the crypts.

BARCLAY J. BARON.

### THERAPEUTICS.

**Enteroclysis or irrigation of the large intestine** in the treatment of summer diarrhœa in children does not seem, if one may

[1] *Med. Chron.*, 3rd Ser., 1899, i. [2] *J. Boston Soc. M. Sc.*, 1898–99 iii. 63.

